# The Impact of Coloring Additives on Thermal, Mechanical, and Tribological Properties of FDM-Printed Components

**DOI:** 10.3390/polym18070855

**Published:** 2026-03-31

**Authors:** Nicoleta Bacescu, Doina Frunzaverde, Vasile Cojocaru, Gerlinde Iuliana Rusu, Raul-Rusalin Turiac, Costel-Relu Ciubotariu, Gabriela Marginean

**Affiliations:** 1 Department of Engineering Science, Babeș-Bolyai University, Traian Vuia Square 1–4, 320085 Reșița, Romania; nicoleta.bacescu@ubbcluj.ro (N.B.); raul.turiac@ubbcluj.ro (R.-R.T.); relu.ciubotariu@ubbcluj.ro (C.-R.C.); 2 Faculty of Chemical Engineering, Biotechnology and Environmental Protection, Politehnica University of Timișoara, Victoriei Square 2, 300006 Timișoara, Romania; gerlinde.rusu@upt.ro; 3 Institute of Mechanical Engineering, Westphalian University of Applied Sciences Gelsenkirchen Bocholt Recklinghausen, Neidenburgerstr. 43, 45897 Gelsenkirchen, Germany; gabriela.marginean@w-hs.de

**Keywords:** polylactic acid (PLA), fused deposition modeling (FDM), filament color, color additives, mechanical properties, ultimate tensile strength (UTS), friction behavior, abrasive wear, thermogravimetric analysis (TGA), differential scanning calorimetry (DSC)

## Abstract

This study examines how manufacturer-specific additive formulations used to obtain nominally identical black PLA filaments influence the thermal, mechanical, and tribological performance of FDM-printed parts. Five commercial filaments were analyzed under identical processing conditions using thermogravimetric analysis (TGA), differential scanning calorimetry (DSC), tensile testing, pin-on-disc measurements, and stereomicroscopy. The filaments exhibited substantial compositional variability, with total additive contents ranging from 2.08 wt.% to 27.82 wt.%. One filament (M5) contained a significant fraction of inorganic fillers, confirmed by SEM/EDX as Ca-, Na- and Mg-based oxides and silicates, identifying it as a PLA-based composite despite being marketed as standard PLA. These differences strongly affected thermal behavior (Tg, Tcc, Tm) and translated directly into the performance of the printed parts. Ultimate tensile strength varied by 88.91% across all filaments (19.38–36.61 MPa), but only by 13% among the four conventional PLA filaments (M1–M4). Tribological performance differed markedly: mean coefficients of friction ranged from 0.246 (M3) to 0.368 (M2), a spread of approximately 50%, with wear-track morphologies reflecting the frictional response. Overall, the results show that PLA filaments cannot be treated as interchangeable materials. Greater transparency and standardized reporting of filament composition are needed to ensure reproducibility and support informed material selection in FDM applications.

## 1. Introduction

Additive manufacturing (AM), or 3D printing, is a major technological advancement that enables layer-by-layer fabrication of components from polymers, metals, and ceramics [1]. Initially developed for rapid prototyping, AM now supports applications across manufacturing, industrial design, medicine, architecture, and related sectors [2]. Its ability to produce customized products, complex prototypes [3], and even biological tissues highlights its growing influence. AM accelerates the conversion of digital concepts into functional objects, enables on-demand production, reduces lead times and costs, and allows the fabrication of geometries unattainable with conventional methods. In medicine, AM provides patient-specific prosthetics and implants and supports progress in bioprinting [4]. Moreover, because additive processes use only the required material, they can reduce waste and energy consumption compared to subtractive techniques.

Fused Deposition Modeling (FDM) is one of the most widely adopted AM methods due to its simplicity and cost-effectiveness [5]. It produces components directly from digital models without molds or machining, minimizing production time and material loss. The low cost of printers and thermoplastic filaments has promoted their adoption in industrial, educational, and hobbyist settings. FDM is used for rapid prototyping, customized part fabrication, tooling, and medical device production.

A key advantage of FDM is the broad range of compatible materials, including engineering thermoplastics and reinforced composites such as carbon-fiber-enhanced polymers. Although material development is advancing, current FDM use remains centered on thermoplastic extrusion. Polylactic acid (PLA) [6,7], a renewable polymer derived from corn starch or sugarcane, is widely used and available in many colors. PLA is biodegradable, emits no toxic fumes during printing, and has a low glass transition temperature. Most PLA grades soften at 60–65 °C, losing rigidity while remaining solid [8]. This behavior can limit use in warm environments but also allows extruded PLA to relieve internal stresses during cooling.

FDM is a versatile yet complex AM technique in which part quality depends strongly on the selection and optimization of process parameters. Numerous studies have shown that printing temperature [9,10,11,12], build-plate temperature [13], build orientation [14,15], layer thickness [16,17], printing speed [6,18,19], raster angle [20], and filament color [16,21,22] significantly influence both the mechanical performance and the aesthetic or functional characteristics of printed parts.

However, additional variables are often underestimated despite their substantial impact [23]. The type and quantity of color additives, filament manufacturer, and printer model can alter polymer thermal behavior, layer adhesion, and even overall part durability. Differences in filament origin and quality may lead to measurable variations in strength and long-term performance, while printer-specific characteristics can introduce further variability even under identical process settings.

Recent studies demonstrate that coloring additives markedly influence the properties of PLA filaments used in FDM printing. Pandzic et al. [24] examined 13 PLA colors and reported substantial variations in Young’s modulus, yield strength, tensile strength, and hardness. Similarly, Iwko et al. [25] found mechanical property differences of up to 45% across colors, emphasizing the relevance of pigment choice. Frunzaverde et al. [9,16] showed that additives affect not only tensile strength but also dimensional accuracy.

Wear behavior is likewise impacted. Research on wear resistance [9,26,27] indicates that filament color plays an important role, with Hanon et al. [26] noting that black pigments reduce wear resistance compared to white and gray samples.

These effects arise because color additives modify PLA’s thermal and structural behavior. Prior work [28,29] shows that pigments can alter melting point and tensile strength, while Vicat softening temperature tests [30] reveal pigment-dependent thermal differences. These findings align with results by Spina [22] and Wittbrodt et al. [31], who identified strong correlations between filament color, printing temperature, and crystallinity—factors collectively influencing tensile strength.

Overall, the chemical composition, particle size, and distribution of pigments within the polymer matrix represent critical factors influencing mechanical performance and thermal stability of printed PLA components [25,32,33,34].

Moreover, manufacturers source raw materials from different suppliers, resulting in variations in polymer properties and distinct PLA grades with differing mechanical behavior. Hodžić et al. [35] compared white PLA filaments from five manufacturers and reported tensile strength variations of up to 17%, with PM Filament reaching 62.7 MPa and 3D Republika only 52.9 MPa under identical printing conditions. Likewise, Andronov et al. [36] evaluated eight commercial white PLA filaments and found that higher price did not consistently correlate with better mechanical properties, dimensional accuracy, or print quality, underscoring the importance of filament origin.

Schwartz et al. [37] examined nine PLA colors from one manufacturer and additional black filaments from two others (20% infill) and showed that additives significantly influence polymer characteristics—including molecular weight, molecular weight distribution, melting enthalpy, and crystallinity—which in turn affect flexural strength, stiffness, and toughness of printed parts.

As pointed out above, recent studies show that the color of PLA filaments affects the dimensional accuracy and material properties of FDM-printed parts, indicating that color must be considered during process optimization. This raises an important supplementary question: can the performance of parts printed with different colors be directly compared—for example, stating that color X ensures the best accuracy, color Y yields superior strength, or color Z provides superior surface quality—or must results be explicitly tied to PLA colors X, Y, Z from a specific manufacturer, acknowledging that identical colors from other manufacturers may yield different outcomes? Progress is limited by insufficient manufacturer transparency and/or standardization regarding filament composition, particularly the type and amount of color additives, hindering accurate performance prediction.

This study addresses the questions outlined in the preceding paragraph by extending previous research, which has typically focused on white/neutral PLA, low infill densities (e.g., 20%), or different mechanical properties. Colored PLA filaments were intentionally selected to evaluate whether the addition of pigments amplifies the performance differences observed between materials. We compared tensile strength and friction behavior of FDM-printed black PLA filaments sourced from five manufacturers. All specimens were fabricated with 100% infill using the same equipment and identical process parameters that complied with each manufacturer’s recommendations. After testing, the structural features of the tensile specimens were examined by stereomicroscopy, while the surfaces and wear tracks of the pin-on-disc samples were characterized using confocal laser scanning microscopy. To clarify the origins of the observed performance differences, the additive content of the five PLA filaments was evaluated by thermogravimetric analysis (TGA) and scanning electron microscopy (SEM) and the thermal behavior of the five filament types was further analyzed through differential scanning calorimetry (DSC).

## 2. Materials and Methods

To investigate the influence of color additives on the tensile strength and abrasive wear behavior of PLA prints manufactured by FDM, black PLA filaments with 2.85 mm diameter were purchased from five randomly selected manufacturers. Since the purpose of this research was not to compare specific manufacturers with one another, but rather to demonstrate that they use different base materials and additives to produce filaments of the same color—differences that can lead to significantly distinct properties in the printed products—the manufacturers were anonymized and the PLA filaments labeled as M1–M5.

From each filament type, five ISO 527-2 1A [38] “dog-bone” tensile specimens with a nominal thickness of 3 mm and two prismatic samples for pin-on-disc testing, measuring 30 × 30 × 10 mm, were fabricated.

Printing was carried out on an Ultimaker 3 FDM printer (Ultimaker BV, Utrecht, The Netherlands) with a fully enclosed build chamber (215 × 215 × 330 mm).

The process parameters applied for the printing of the 35 tensile testing specimens and respectively 10 prismatic pin-on-disc samples are listed in Table 1. Their values fall within the ranges recommended by each filament supplier. All samples were fabricated individually—one per build plate—in the YX orientation, following the specifications of ISO/ASTM 52921:2013 [39]. Neither the filament nor the printed specimens underwent any pre- or post-processing. Before printing, the PLA spools were kept in sealed packaging to minimize moisture uptake and prevent exposure to UV radiation.

Tensile tests were performed using an Instron 5584 electromechanical universal testing machine (Instron, Norwood, MA, USA), equipped with a 150 kN load cell. The system features a dual-column test frame with a maximum crosshead travel opening of 52 in and a test speed range of 0.002–20 in/min. All measurements were realized in accordance with ISO 527-1 [40] and ISO 527-2 [38], using a constant crosshead speed of 10 mm/min. For each specimen group, the ultimate tensile strength (UTS) was determined, along with the corresponding standard deviations.

The mesostructures of the fractured tensile specimens were examined on both the fracture surfaces and the top-layer surfaces using a Leica MZ 7.5 stereomicroscope (Leica Microsystems, Wetzlar, Germany) at a magnification of 12.5×.

The abrasive wear behavior of the samples was investigated using a pin-on-disc apparatus, specifically a CSM tribometer (CSM Instruments Tribometer, Needham, MA, USA) in accordance with ISO 7148-2:2012(E) [41]. The stationary counterpart was an Al_2_O_3_ (aluminum oxide) ball with a diameter of 6 mm. The wear track radius was set to 7 mm, the applied normal load to 10 N, and the sliding speed was maintained at 150 mm/s. The tests were carried out at a temperature of 23 °C and a relative humidity of 50%. For each manufacturer, two samples (30 × 30 × 10 mm) were tested, and the mean coefficient of friction for each pair was determined based on the average values provided by the testing instrument. Prior to testing, both the alumina ball and the sample surfaces were cleaned with ethanol. Each specimen was subjected to 10,000 cycles. Subsequently, Keyence confocal laser scanning microscope type CLSM (Keyence VKX-260K, Neu-Isenburg, Germany) was employed to evaluate the surface features and wear tracks.

To determine the number and quantities of additives incorporated by the manufacturers into the five black PLA filaments, thermogravimetric analysis (TGA) was performed using an STA 449 F1 Jupiter NETZSCH analyzer (Netzsch-Gerätebau GmbH, Selb, Germany). The measurements were carried out in nitrogen and synthetic air atmosphere, starting from an initial temperature of 30 °C and heating up to 1000 °C at a constant rate of 15 °C per minute.

The thermal properties of the five types of black PLA filaments were assessed by means of differential scanning calorimetry in nitrogen atmosphere (N_2_, 20.0 mL/min) using a DSC 204 F1 Phoenix calorimeter (Netzsch-Gerätebau GmbH, Selb, Germany), which ensures a temperature measurement uncertainty of 0.1–0.3 °C. The testing conditions were as follows: temperature between 30–230 °C, heating rate 10 °C/min. To prevent pressure buildup during any potential evaporation, the samples were weighed in sealed aluminum crucibles fitted with perforated lids. Thermograms were analyzed using Netzsch Proteus Thermal Analysis version 6.1.0 software.

## 3. Results and Discussions

The experimental results, illustrating the influence of color additives on the tensile strength and friction behavior of black PLA specimens manufactured by FDM, are presented and discussed in the following subsections. To facilitate the visual distinction of manufacturer-specific characteristics, all graphical representations employ a consistent color scheme: red for M1, mustard yellow for M2, black for M3, green for M4, and blue for M5.

For the beginning, the five types of filaments were subjected to thermogravimetric analysis (TGA), scanning electron microscopy (SEM), and respectively differential scanning calorimetry (DSC) in order to characterize their composition and thermal properties.

### 3.1. Thermogravimetric Analysis (TGA)

**Figure 1:**
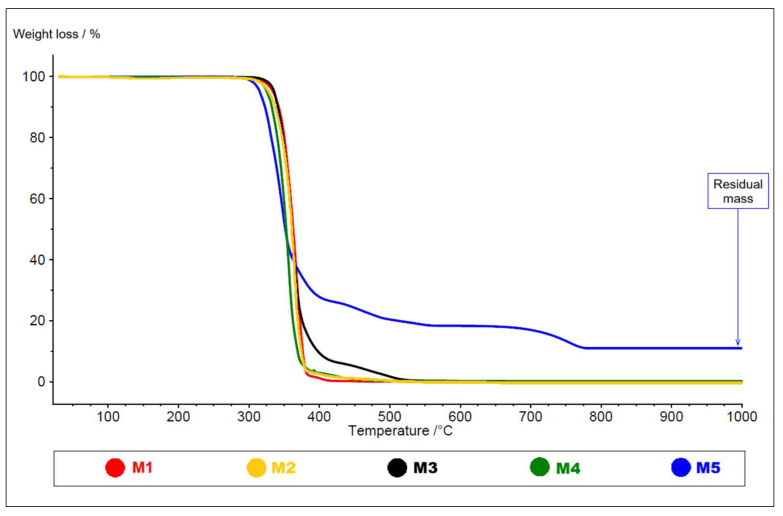
TGA curves for the black PLA filaments M1–M5, sourced from different manufacturers.

Thermogravimetric analysis revealed clear manufacturer-dependent differences in both composition and thermal stability for the black PLA filaments sourced from different manufacturers, as presented in Figure 1.

The detailed results of the TG analysis—including the onset degradation temperature, the number of additives, and their respective contents—are presented in Table 2. Additives decomposing between 350 °C and 800 °C (Additives 1–3) and those remaining as residual mass at 1000 °C are both reported. The numbering of the additives (Additives 1–3) reflects exclusively the order in which they decomposed during the TG analysis, at temperatures specific to each filament (M1–M5), and does not correspond to any particular additive type common to all five filaments.

As presented in Table 2, all samples were shown to contain more than one additives. Filaments M1–M4 showed near-complete burning off with negligible residual mass, indicating low inorganic loading and total additive contents between 2–6.5 wt.% (M1–2.08%; M2—5.00%; M3—6.48%; M4—3.46%), whereas the filament M5 exhibited a markedly higher additive fraction (27.82 wt.%) and a substantial final residue at about 1000 °C (10.97 wt.%), consistent with significant inorganic pigment/filler content.

These compositional differences were accompanied by shifts in the onset of thermal degradation: filaments M1–M3 initiated major mass loss above 343–347 °C (M1—at 347 °C, M2—at. 347 °C, and M3—at 343 °C), while the filaments M4 and M5 started degradation at notably lower temperatures (M4—at 337 °C and M5—at 320 °C), evidencing reduced thermal stability.

Importantly, the unusually high inorganic content and reduced thermal stability of filament M5 indicate that it is not a standard PLA filament but a PLA-based composite material. This finding is noteworthy because M5 is not marketed as a composite, and no such information is disclosed by the manufacturer. For this reason, M5 was retained in the study not as a member of the M1–M4 group but as a distinct, illustrative case, emphasizing the broader issue that end users cannot obtain predictable or comparable material performance when manufacturers do not transparently report filament compositions. The behavior of M5 therefore highlights a systemic knowledge gap affecting reproducibility in FDM materials research.

### 3.2. Scanning Electron Microscopy (SEM)

**Figure 2:**
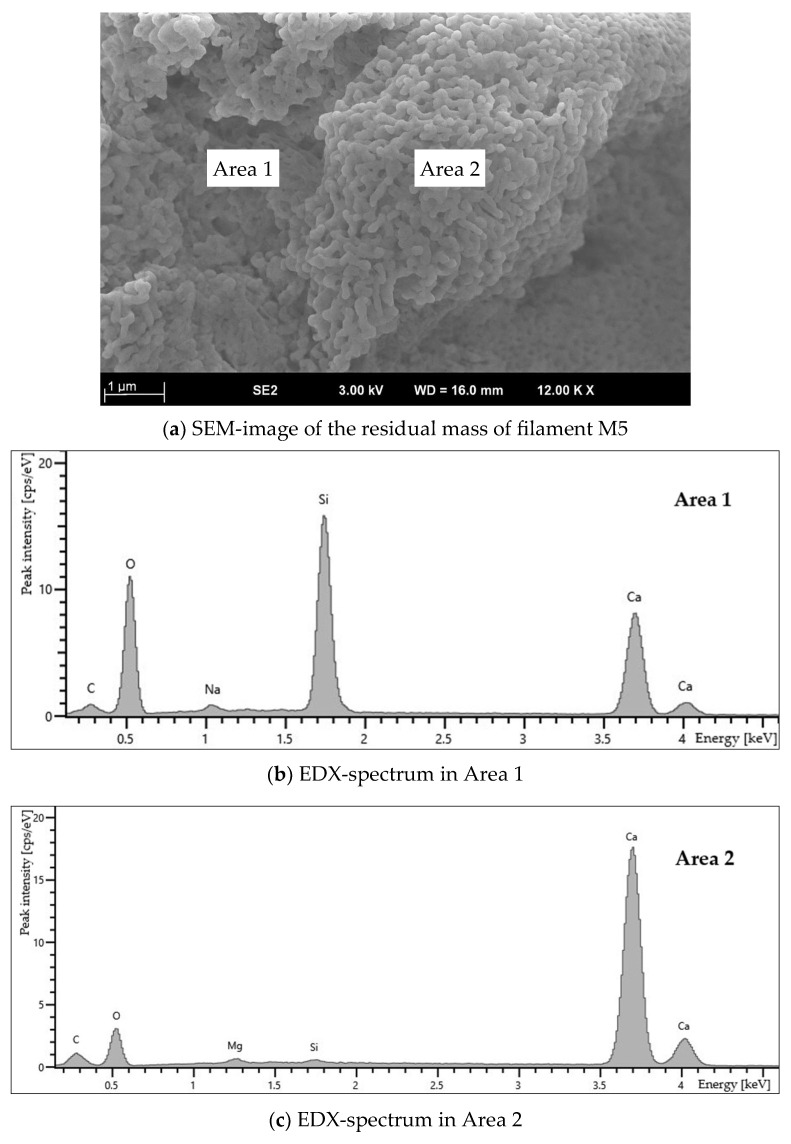
SEM-image (**a**) and EDX-spectra (**b**,**c**) of the residual mass of filament M5.

To identify the composition of the residual mass found in the M5 filament, scanning electron microscopy and energy-dispersive X-ray spectroscopy (EDX) were performed. The results of these analyses are presented in Figure 2.

As shown by the EDX-spectra (Figure 2b,c), the high-temperature-stable residue of the PLA-based composite material (M5) consists of oxides and silicates containing Ca, Na, and Mg. The SEM image of the residue reveals the presence of smooth or slightly rough submicron particles, which are necked together into agglomerates (Figure 2a).

### 3.3. Differential Scanning Calorimetry (DSC)

**Figure 3:**
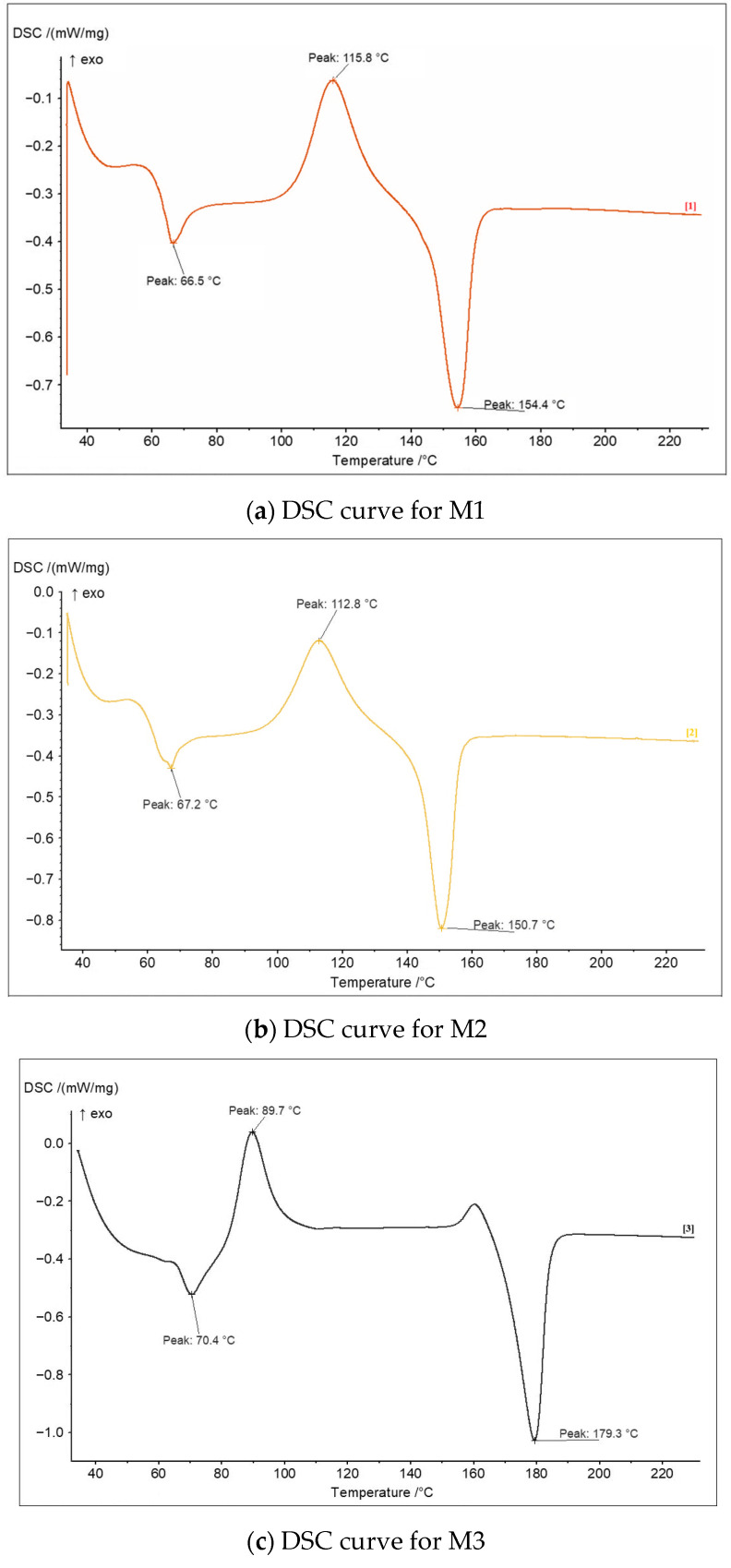

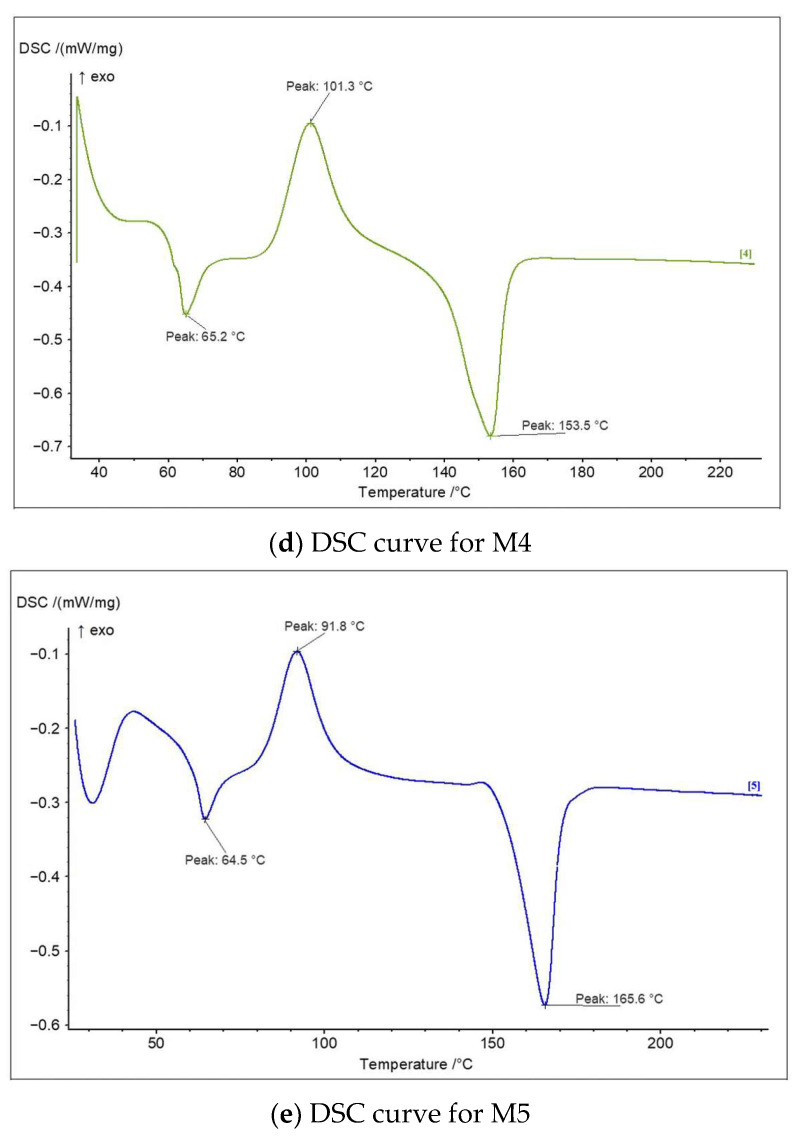
DSC curves for the black PLA filaments sourced from different manufacturers.

The DSC curves registered for the five black filament types are presented in Figure 3. They highlight differences among the PLA filaments sourced from different producers, reflecting the influence of manufacturer-specific compositions and additive systems.

As shown by the thermograms in Figure 3, all materials exhibit the characteristic thermal transitions of PLA: glass transition (Tg), cold crystallization (Tcc), and melting of the crystalline phase (Tm). In all samples, the glass transition displays a peak-like endothermic overshoot superimposed on the heat-capacity step. This behavior is typical for polymers stored below Tg and arises from enthalpic relaxation (physical aging). During storage, the amorphous phase gradually evolves toward a more ordered, non-equilibrium state. Upon subsequent heating through Tg, the polymer relaxes back toward thermodynamic equilibrium, producing an enthalpic recovery peak. Such peak–step behavior is well documented in DSC application notes from TA Instruments [42,43] and is consistent with the kinetic interpretation of the glass transition provided by Argatov and Kocherbitov [44], who demonstrate that enthalpy relaxation can generate overshoot features during constant-rate DSC heating.

However, both the temperatures and shapes of these transitions vary noticeably among the filaments M1–M5, consistent with prior observations that compositional variables (e.g., inorganic fillers, nucleants, colorants) modulate chain mobility and crystallization pathways in PLA [45,46,47]. Overall, filaments M1, M2, and M4 exhibit similar thermal behavior, while M3 shows an earlier onset of cold crystallization together with a higher melting temperature, reflecting a more stable and well-organized crystalline structure. In contrast, the composite filament M5 presents an intermediate thermal profile.

For ease of comparison, the DSC results are also summarized in Table 3. The glass transition temperatures (Tg) ranged from 64 °C for the composite material M5 to 70 °C for filament M3. Among the colored PLA filaments (M1–M4), Tg values varied between 64 °C (M4) and 70 °C (M3), corresponding to a difference of approximately 8%. The filament M3 showed the highest Tg (70 °C), indicating increased molecular rigidity or a lower degree of plasticization, whereas the M 5 composite exhibited the lowest Tg (64 °C), consistent with the elevated additive content revealed by the TGA (Table 2) and prior research [45,46,47], showing that mobility-enhancing additives (e.g., plasticizers/pigments or poorly interacting particulates) generally depress Tg, whereas more homogeneous or nucleated matrices tend to display higher Tg due to restricted mobility or more uniform chain packing.

Beyond Tg, the sharpness and position of Tcc and Tm also differ among filaments. The filaments M1–M3 display sharper (narrower) Tcc/Tm features, indicative of a relatively uniform molecular environment and a narrower distribution of crystallizable domains—behavior widely reported when nucleating agents or well-dispersed fillers promote more coherent ordering and reduce the temperature window for crystallization [46]. By contrast, the broadened Tcc and Tm observed for the composite material M5 are consistent with inhibited and heterogeneous crystallization arising from a higher additive load and/or pigment/filler effects that disrupt lamellar perfection and spread crystallization/melting over a wider temperature range, as also revealed by previous research [45,47]. These trends are in line with studies showing that inorganic fillers and certain bio-based modifiers can shift Tg and alter Tcc/Tm through changes in nucleation density, interfacial interactions, and thermal history. Complementary thermo-analytical work on PLA also documents how degradation/structural changes influence the apparent DSC transitions and their breadth [45,46,47,48].

Furthermore, based on the melting enthalpy values (ΔH, Table 3)—which, consistent with previous findings [49,50], reflect the crystallinity level of PLA materials—it can be inferred that sample M3 exhibits the highest degree of crystallinity, sample M5 (composite material) the lowest, while M1, M2, and M4 display intermediate crystallinity.

Overall, the DSC analysis confirmed that the thermal behavior of PLA is strongly influenced not only by the presence of fillers (as in M5), but also by the type and content of coloring additives. These thermal differences manifested directly in the mechanical and tribological performance of the printed components, as shown below.

### 3.4. Tensile Behavior

**Figure 4:**
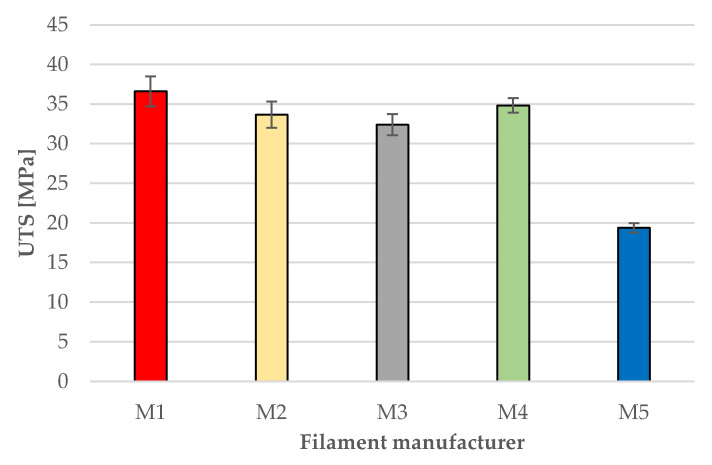
The ultimate tensile strength (UTS) of the samples printed with black PLA filaments sourced from the 5 different manufacturers (M1–M5).

Figure 4 presents the mean values of the ultimate tensile strength (UTS), together with the associated standard deviations, for the specimens produced from black PLA filaments sourced from the five manufacturers. As previously mentioned, each manufacturer was represented by a set of five tensile specimens.

As shown in Figure 4, the average tensile strength values ranged from 19.38 MPa for the composite material M5 to 36.61 MPa for filament M1. The difference between these mean values is substantial—88.91%—yet consistent with the pronounced compositional differences between the materials.

With the exception of the pronounced outliner (composite material M5), the remaining four sets of specimens exhibited more comparable tensile strength values, ranging between 32.40 MPa (M3) and 36.61 MPa (M1). Nevertheless, even in this case, the difference between the extreme values is approximately 13% (12.98%).

The combined TGA, DSC, and tensile results reveal clear structure-property relationships consistent with previously reported behavior of PLA-based materials. The TGA showed that filaments M1–M3 exhibited the highest onset degradation temperatures (343–347 °C), indicating a more thermally stable PLA matrix with fewer thermally labile additives, whereas M5 displayed pronounced thermal instability attributable to its exceptionally high additive content (27.82 wt%). Such reductions in thermal stability are well-known to weaken PLA mechanical performance because degraded chains exhibit lower entanglement density and inferior load-bearing capacity, as highlighted in comprehensive structure–property reviews of PLA [51].

The DSC data further clarify these differences: M1, M2, and M4 exhibit moderate melting enthalpy values (ΔH ≈ 23–25 J/g), corresponding to intermediate crystallinity, whereas M3 shows the highest crystallinity (ΔH = 46 J/g), and M5 the lowest (ΔH = 19 J/g). These results are consistent with studies showing that increased crystallinity-whether induced by drawing, annealing, or optimized thermal processing-generally enhances tensile performance by promoting tighter chain packing and more coherent lamellar development [52,53]. However, excessive crystallinity may also increase brittleness, explaining why M3, despite its more developed crystalline structure, does not achieve the highest UTS among the five filaments. Noh et al. [52] demonstrated that crystallinity improves tensile strength only up to the point where highly oriented crystalline domains begin to promote premature necking or brittle failure during tensile loading.

In contrast, the weak thermal signature of the composite filament M5—lower Tonset, broadened Tcc and Tm peaks, and reduced crystallinity—reflects a heterogeneous and poorly organized molecular structure strongly influenced by its high pigment/filler loading. Prior studies on PLA composites have shown that such disruptions in nucleation and crystal growth significantly diminish mechanical strength due to impeded lamellar formation and reduced matrix continuity [54]. These thermal deficiencies directly correspond to its markedly reduced UTS (19.38 MPa), far below the 32–37 MPa range observed for the other filaments. Overall, these findings align with existing literature showing that PLA tensile behavior is governed by the interplay between crystallinity, thermal stability, and the presence of structural modifiers, all of which collectively determine chain mobility, crystal formation, and the capacity of the polymer network to sustain tensile loads [51,52,53].

### 3.5. Mesostructure of the PLA Samples

**Figure 5:**
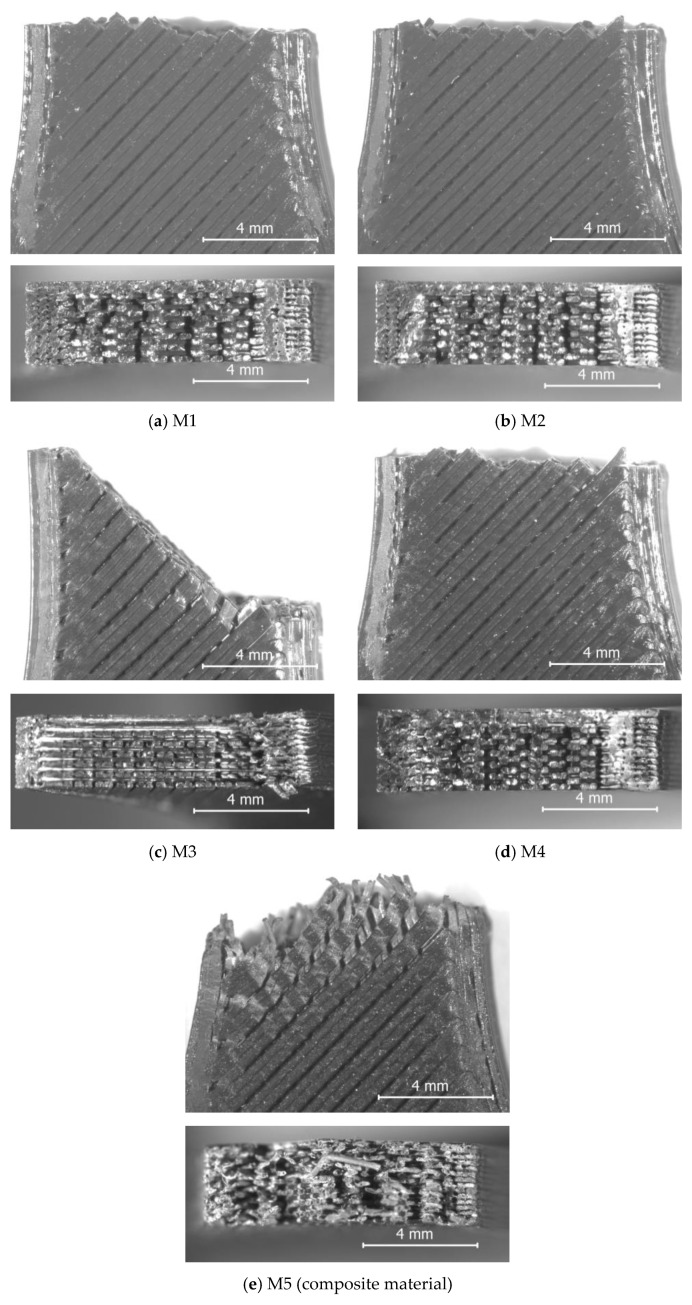
Top view (**up**) and fractured surface (**down**) of the tensile specimens fabricated with black PLA sourced from different manufacturers (M1–M5). Stereomicroscopic images. Magnification: 12.5×.

To elucidate the factors underlying these results, after tensile testing, both the surface morphology and the fracture cross-section of the specimens were examined using stereomicroscopy, as described in Section 2. Representative micrographs for each batch of specimens are shown in Figure 5.

In FDM printing, UTS is strongly governed by inter-road and respectively inter-layer bonding, which depends on melt wetting and polymer chain interdiffusion (“thermal welding”) before the interface vitrifies and/or crystallizes, while defects such as voids/lack-of-fusion act as dominant stress concentrators [55,56].

As presented in Section 3.5, the UTS values of the FDM printed black PLA filaments span between 19.38–36.61 MPa, with M1–M2–M4 forming a high-strength cluster (≈34–37 MPa), M3 achieving a slightly lower strength (≈32.4 MPa), and M5 a clear outlier (19.38 MPa) (see Figure 4). The stereomicroscopic fracture surfaces corroborate these rankings: M1, M2, M4 failed predominantly by straight, load-normal fracture (Figure 5a,b,d), typical for brittle tensile failure in adequately welded FDM laminates, whereas M3 (Figure 5c) and M5 (Figure 5e) exhibited inclined, shear-dominated fracture planes. This is consistent with FDM fracture mechanics where inter-road and interlayer diffusion (“thermal welding”) governs strength and failure mode [55,56]. Prior studies likewise report that reduced interlayer bonding and defect populations shift failure toward shear-dominated delamination in PLA FDM [9,16,22,34].

As illustrated in Figure 5, the mesostructural consolidation quality observed by stereomicroscopy is consistent not only with the UTS-ranking, but also with the combined thermal–compositional evidence from the TG/DSC analyses.

Thus, the TG results indicated that the PLA filament from M5 is a highly compounded filament, containing 27.82 wt.% total additives and an important filler fraction (10.97 wt.% residue), whereas filaments provided by M1–M4 contain only 2.08–6.48 wt.% additives and left no residue. In parallel, M5 exhibited the lowest thermal stability (lowest Tonset = 320.8 °C, vs. 337.5–347.4 °C for M1–M4, as well as lowest Tg = 64.4 °C, vs. 65.20–70.40 °C). Under same printing conditions (nozzle temperature 210 °C, bed temperature 60 °C), the existing composition- and thermal-response differences translated into differences of the melt flow/wetting and the effective interdiffusion time window, which, together with the higher probability of particulate-rich defects, rationalize the poor mesostructural continuity (Figure 5e) and the strongly reduced UTS (19.38 MPa) of the M5 samples. These effects are consistent with those reported also by previous research [55,56,57].

Conversely, M1—the highest-strength filament (36.61 MPa)—combines the lowest additive content (2.08 wt.%) with comparatively high thermal stability and the highest Tcc (115.8 °C), consistent with delayed crystallization and a longer diffusion (“welding”) window during cooling, which promotes stronger inter-road bonds and higher UTS, as also noted by other authors [55,56]. Consequently, the corresponding mesostructure (Figure 5a) reveals adequate consolidation.

Within the M1–M4 group, the lower UTS of M3 aligns with its distinct DSC profile (highest Tg = 70.4 °C, lowest Tcc = 89.7 °C, smallest diffusion window = 19.3 °C), suggesting a different crystalline population that may reduce bonding efficiency at fixed processing settings, in agreement with the findings of [45,58] and the poor mesostructural consolidation revealed in Figure 5c for this otherwise low-additive filament.

### 3.6. Friction Behavior

**Figure 6:**
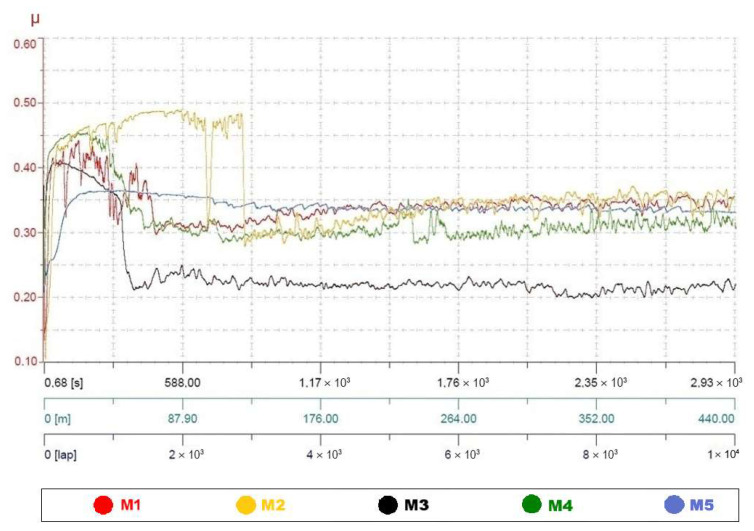
Variation in the friction coefficients during the pin-on-disc test for specimens fabricated with black PLA sourced from different manufacturers (M1–M5). The vertical axis represents the friction coefficient (μ), while the horizontal axis indicates the test duration (up to 2930 s), sliding distance (up to 440 m), and number of laps (up to 10,000 laps).

The five types of black PLA were also compared in terms of their abrasive wear behavior. For each manufacturer, two specimens were tested using the pin-on-disc tribometer. In all cases, the test results for the two specimens belonging to the same batch were highly similar. For this reason, for each filament type, a single representative curve, illustrating the variation of the coefficient of friction throughout the experiment, was selected for the comparative analysis. To visually compare the friction behavior of the filaments from different manufacturers, these curves were superimposed into a single graphical representation (Figure 6).

As one can see in Figure 6, for all five black PLA filament types, the variation in the coefficient of friction (COF, μ) during the pin-on-disc test evolved in two main stages, respectively a running-in stage followed steady-state regime. However, the duration of the run-in stage, as well as the stability and magnitude of the coefficient of friction during the two stages, differ considerably across manufacturers.

Regardless of the stage, the curves consistently fell between those of the filament M2 and those of the filament M3. Thus, M3 consistently exhibits the lowest friction coefficients and the most stable sliding, with the shortest running-in period. In contrast, M2 presents the highest COF values, the greatest fluctuations, and the most unstable frictional response throughout the test. The other filaments (M1, M4, M5) fall between these extremes, with M5 presenting a notably long running-in stage but relatively stable steady-state friction. Overall, the friction analysis shows that the tribological performance of PLA is highly manufacturer-dependent, even when filament color and printing conditions are identical.

**Figure 7:**
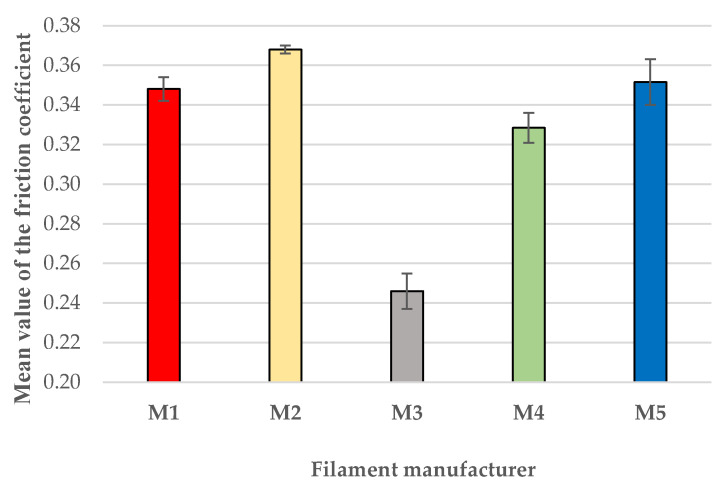
Mean values of the average friction coefficients registered for each batch of samples M1–M5. Error bars are repeatability-based error bars.

In order to allow for a quantitative comparison between the friction performances of the PLA prints, the arithmetic mean of the average friction-coefficient values experimentally determined by the pin-on-disc tester for each batch of two samples printed with the same filament (M1–M5) was calculated and plotted in Figure 7. Although only two specimens were employed per material, the corresponding repeatability bands were sufficiently small to ensure acceptable measurement reliability, with values of ±0.0060 (M1), ±0.0020 (M2), ±0.0090 (M3), ±0.0075 (M4), and ±0.0115 (M5). These uncertainty levels are directly visualized in Figure 7 through the repeatability-based error bars, confirming that the observed differences between materials exceed the measurement scatter. In addition, as described below, 3D confocal laser-scanning microscopy of the wear tracks revealed highly similar wear profiles within each pair of specimens, further supporting the consistency of the duplicate tests. As the objective of this study was to compare tendencies among the tested filaments rather than to establish precise quantitative friction values, the combination of acceptable repeatability, coherent COF profiles, and comparable wear morphologies provides confidence that the significant differences observed between the filament types are meaningful and representative.

So, as already pointed out above, the lowest coefficient of friction was recorded for the samples produced using the M3 filament, with an average COF value of 0.246. In contrast, the samples manufactured with the M2 filament showed the highest values of the COF, respectively, 0.368. The difference between the extreme values (M3 vs. M2) is 50% (49.59%), confirming significant manufacturer-dependent variations even for filaments of the same nominal material (black PLA) printed under identical conditions. It may be mentioned that all values of the coefficient of friction fall into the typical COF interval, ranging from 0.2–0.4, mentioned by previous research [9,59,60] for dry sliding of FDM-printed PLA against steel, at moderate normal loads (5–20 N) and room temperature.

The differences in COF among the PLA filaments (Figure 7) can be directly linked to their thermal behavior. Filament M3, which showed the highest crystallinity (ΔH = 46 J/g) and good thermal stability (Tonset = 343 °C), also exhibited the lowest and most stable COF. Higher crystallinity typically strengthens lamellar structures, reducing adhesive interactions and stabilizing sliding, as reported for semicrystalline polymers including PLA and UHMWPE [61,62]. In contrast, M2—despite its high Tonset (347 °C)—contains a high pigment/additive load (5%), which likely disrupted crystalline ordering and led to the highest COF and the most unstable frictional response, consistent with findings showing that fillers and pigments increase interfacial shear and friction variability [61,63]. The composite filament M5, with its very low crystallinity and extremely high additive content, showed a long running in period and intermediate COF, a behavior aligned with studies reporting that heterogeneous crystallization and extensive filler loading increase micro ploughing and wear debris formation [64]. Overall, the COF results consistently reflect the combined influence of thermal stability, crystallinity, and additive distribution, confirming that the tribological performance of PLA is strongly governed by the thermal and morphological features established during processing.

**Figure 8:**
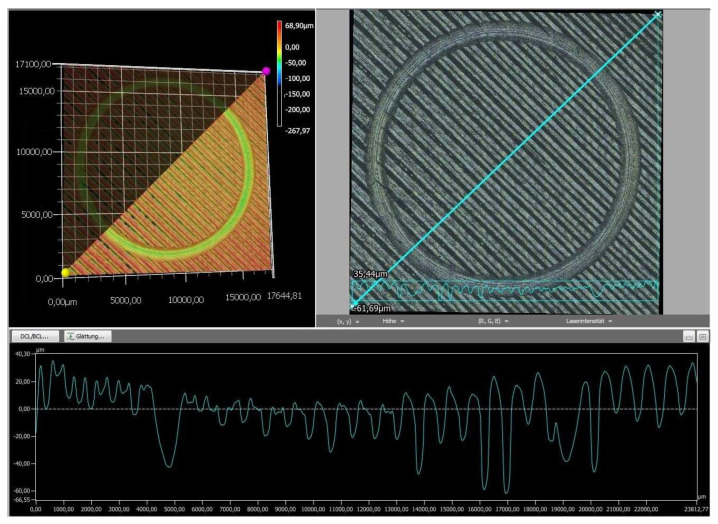
The wear profile of the test specimen printed with the M1 filament.

**Figure 9:**
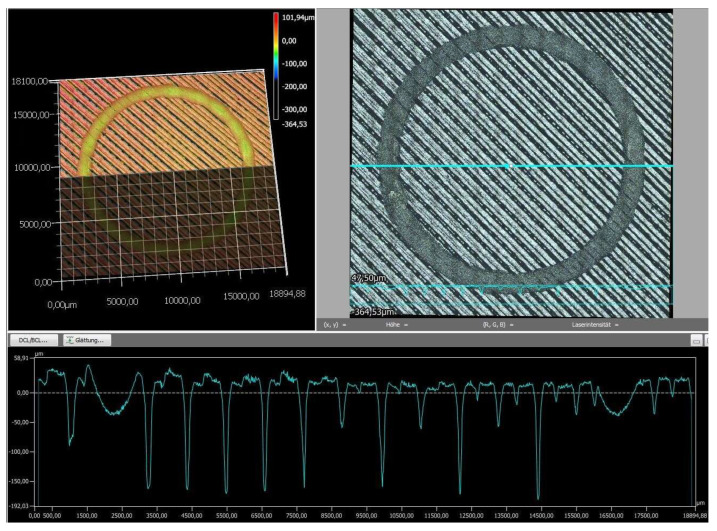
The wear profile of the test specimen printed with the M2 filament.

**Figure 10:**
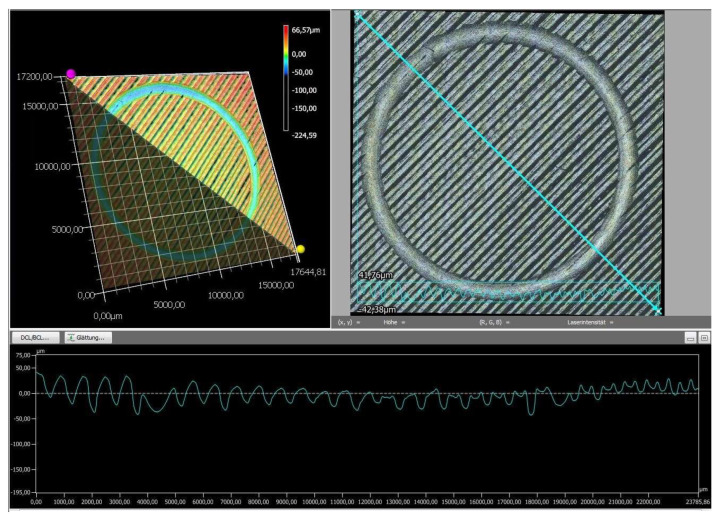
The wear profile of the test specimen printed with the M3 filament.

**Figure 11:**
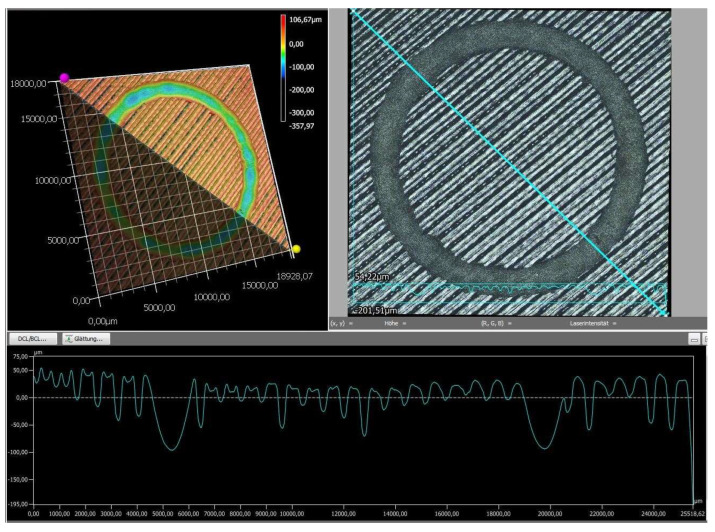
The wear profile of the test specimen printed with the M4 filament.

**Figure 12:**
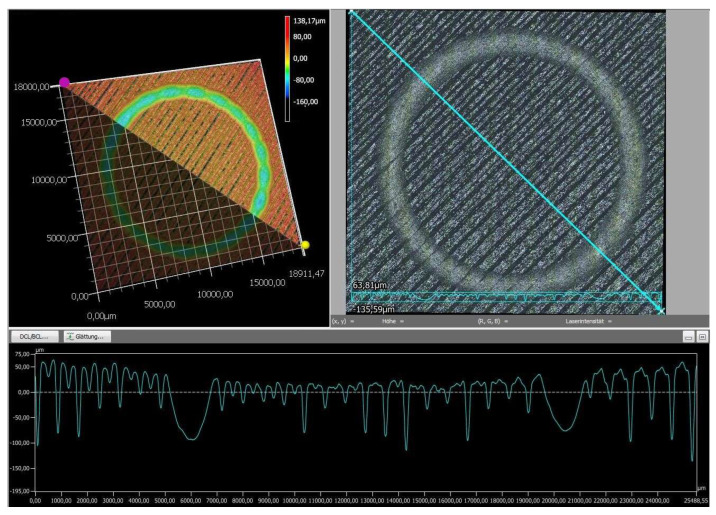
The wear profile of the test specimen printed with the M5 filament.

Finally, the wear tracks of the PLA specimens were examined using digital microscopy to provide a more detailed assessment of their overall wear behavior. The images of the wear tracks and the wear profiles obtained after pin-on-disc testing are shown in Figure 8, Figure 9, Figure 10, Figure 11 and Figure 12.

As revealed by the images above (Figure 8, Figure 9, Figure 10, Figure 11 and Figure 12), the friction responses of the five black PLA filaments, illustrated in Figure 6 and Figure 7, are fully supported by the wear-track morphologies observed in Figure 8, Figure 9, Figure 10, Figure 11 and Figure 12. The sample printed with M3, which exhibited the lowest mean coefficient of friction (0.246) and the shortest running-in period, generated a narrow and shallow wear track with minimal debris (Figure 10), indicating low real contact area and a stable transfer-film formation consistent with its smooth COF profile. In contrast, the sample printed with M2, which recorded the highest friction levels (μ = 0.368) and the largest COF fluctuations, developed the widest and deepest wear track with pronounced material removal and irregularities (Figure 9), confirming the unstable sliding behavior reflected in its friction curve. The intermediate COF values measured for M1 and M4 correspond to moderately worn surfaces (Figure 8 and Figure 11), characterized by limited ploughing and partially uniform textures, aligning well with their mid-range friction plateaus. The sample printed with the M5 composite displayed the longest running-in stage (≈3000 laps) but a stable steady-state COF. Its wear track (Figure 12) shows heterogeneous early damage and debris islands—consistent with delayed surface conditioning—followed by a more uniform tribological contact as the test progressed. Overall, the surface appearances observed for all five materials corroborate the COF profiles and mean values, demonstrating a clear and coherent relationship between the recorded friction behavior and the resulting wear-track morphology.

## 4. Conclusions

This study demonstrated that black PLA filaments marketed as nominally identical materials can exhibit substantial manufacturer-dependent differences in composition and, consequently, in their thermal, mechanical, and tribological performance.

Thermogravimetric analysis showed that the total additive content ranged from 2.08 wt.% to 27.82 wt.%, with filament M5 containing a significant inorganic fraction (identified by SEM/EDX as Ca-, Na- and Mg-bearing oxides and silicates). These results confirmed that M5 is, in fact, a PLA-based composite, despite not being marketed as such. For this reason, M5 was considered separately from M1–M4, highlighting the challenges posed by insufficient transparency regarding filament formulations.

The DSC analysis further showed that differences in additive type and concentration affect PLA’s thermal response, shifting Tg, Tcc, and Tm and altering the sharpness of the transitions.

These thermal variations translated directly into the mechanical behavior of the printed parts. Across the full set of five filaments (M1–M5), the ultimate tensile strength (UTS) varied by 88.91% (19.38–36.61 MPa). When excluding the composite material (M5), the UTS variation among the four conventionally formulated PLA filaments (M1–M4) narrowed to approximately 13%, indicating significant performance differences also within this group.

The tribological results also revealed strong manufacturer dependence. The mean coefficient of friction (COF) values ranged from 0.246 (M3) to 0.368 (M2), representing a substantial spread of approximately 50%, with wear-track morphologies fully consistent with the recorded friction traces. Filaments exhibiting sharper thermal transitions and higher crystallinity produced narrower, smoother wear tracks and more stable sliding, whereas those with higher additive loadings showed wider tracks and extended running-in behavior.

In our representative selection, filaments M1 and M4 provided the most favorable balance of high UTS and acceptable friction behavior. Nevertheless, the goal of this study was not to identify the best-performing filament but to demonstrate that the performance of PLA prints—even when nominally identical in color and processed under the same conditions—depends strongly on the manufacturer’s specific additive package and base-material quality.

Overall, the results emphasize that PLA filaments cannot be assumed to be interchangeable. Differences in pigment chemistry, additive concentration, and the presence of undisclosed fillers can significantly influence thermal stability, crystallization behavior, interlayer bonding, tensile performance, and frictional response. These findings underscore the need for greater transparency and standardized reporting of filament composition to ensure reproducibility, facilitate meaningful comparison across studies, and support informed material selection in FDM applications.

## Figures and Tables

**Table 1:** FDM-process parameters.

Parameters		Values
Fixed process parameters	Printing head temperature, T_H_ [°C]	210
	Build plate temperature, T_B_ [°C]	60
	Layer thickness, t [mm]	0.20
	Nozzle diameter, d_n_ [mm]	0.40
	Filament diameter, d_f_ [mm]	2.85
	Build orientation	YX
	Raster angle, θ [°]	45/−45
	Infill density [%]	100
	Number of wall lines, W_L_ [-]	2
	Number of simultaneously printed samples [-]	1 (individually printing)
	Material/Filament color	Black PLA
	Printing speed [mm/s]	50
Variable parameters	Filament manufacturer	M1–M5

**Table 2:** Results of the TG analyze.

Results	PLA Filament Type				
	M1	M2	M3	M4	M5
Humidity [wt.%]	0.46	0.37	0.15	0.41	0.16
Onset degradation temperature of PLA, Tonset [°C]	347	347	343	338	321
Additive 1 [wt.%]	0.81	3.11	2.72	1.95	6.77
Additive 2 [wt.%]	1.02	1.49	3.76	1.30	2.72
Additive 3 [wt.%]	0.25	0.40		0.21	7.36
Total content of burned off additives [wt.%]	2.08	5.00	6.48	3.46	16.85
Residual mass [wt.%]	0.00	0.00	0.00	0.00	10.97
No. of additives incorporated into the PLA filament (burned off and residual)	3	3	2	3	4
Total content of additives (burned off and residual) [wt.%]	2.08	5.00	6.48	3.46	27.82

**Table 3:** Results of the DSC analysis.

Results	Manufacturer				
	1	2	3	4	5
Glass transition temperatures, Tg [°C]	67	67	70	65	64
Cold crystallization peaks, Tcc [°C]	116	113	90	101	92
Melting peak, Tm [°C]	154	151	179	154	166
Melting enthalpy, ∆H [J/g]	24	25	46	23	19

## Data Availability

The original contributions presented in this study are included in the article. Further inquiries can be directed to the corresponding authors.
